# NORA: An Approach for Transforming Network Management Policies into Automated Planning Problems

**DOI:** 10.3390/s21051790

**Published:** 2021-03-04

**Authors:** Angela Rodriguez-Vivas, Oscar Mauricio Caicedo, Armando Ordoñez, Jéferson Campos Nobre, Lisandro Zambenedetti Granville

**Affiliations:** 1 Department of Telematics, University of Cauca, Popayán 190002, Colombia; omcaicedo@unicauca.edu.co (O.M.C.); jaordonez@unicauca.edu.co (A.O.); 2 Informatics Institute, Federal University of Rio Grande do Sul, Porto Alegre, RS 90040-060, Brazil; jcnobre@inf.ufrgs.br (J.C.N.); granville@inf.ufrgs.br (L.Z.G.)

**Keywords:** WSN, mobile sensors, sweep coverage, approximation algorithm, combinatorial mathematics

## Abstract

Realizing autonomic management control loops is pivotal for achieving self-driving networks. Some studies have recently evidence the feasibility of using Automated Planning (AP) to carry out these loops. However, in practice, the use of AP is complicated since network administrators, who are non-experts in Artificial Intelligence, need to define network management policies as AP-goals and combine them with the network status and network management tasks to obtain AP-problems. AP planners use these problems to build up autonomic solutions formed by primitive tasks that modify the initial network state to achieve management goals. Although recent approaches have investigated transforming network management policies expressed in specific languages into low-level configuration rules, transforming these policies expressed in natural language into AP-goals and, subsequently, build up AP-based autonomic management loops remains unexplored. This paper introduces a novel approach, called NORA, to automatically generate AP-problems by translating Goal Policies expressed in natural language into AP-goals and combining them with both the network status and the network management tasks. NORA uses Natural Language Processing as the translation technique and templates as the combination technique to avoid network administrators to learn policy languages or AP-notations. We used a dataset containing Goal Policies to evaluate the NORA’s prototype. The results show that NORA achieves high precision and spends a short-time on generating AP-problems, which evinces NORA aids to overcome barriers to using AP in autonomic network management scenarios.

## 1. Introduction

Networks’ complexity and size are growing exponentially, making unfeasible their manual administration. The self-driving networks paradigm comes with the promise of accomplishing minimal or null human intervention [1,2]. Realizing autonomic control loops (ACLs) for network management based on Artificial Intelligence (AI) techniques, like Automated Planning (AP) [3], Machine Learning (ML) [4], or their combination, is pivotal for achieving the self-driving networks’ promise. Specifically, AP has been used in the networking domain to create autonomic solutions (or plans) formed by a set of primitive tasks that takes the network from an (troublesome) initial state to a desired state that satisfies network management policies. However, carrying out AP-based ACLs is complicated since network administrators, who are non-AI-experts, need to define network management policies as AP-goals in an AP notation, and combine them with the network status and network management tasks to obtain AP-problems. An AP-problem is a primary input for an AI planner to build up a solution plan.

In several domains like information technologies and telecommunications, diverse approaches [5,6,7] have been proposed to automatically translate policies expressed in natural language (NL) into AP-goals. Nevertheless, as these approaches use translation rules fitted to their domains, their adaptability to other ones is constrained. In the network management domain, some efforts based on policies refinement have been introduced to translate network management policies defined in Controlled Natural Language (CNL) [8,9], Intents [10], or Requirement Formats [11] into Software-Defined Networks (SDN) flow rules [12] or P4 programs [13]. These policies’ refinement-based approaches share some shortcomings. They require policies described in a particular syntax, such as CNL and Intents; overall, these syntax can be as hard to learn and interpret for network administrators as the AP notations. Furthermore, they do not offer an interpretation bridge between management policies and AI notations, hindering AP-problems’ realization and, consequently, the challenge of building up AP-based ACLs for network management remains unexplored.

This paper introduces a novel approach, called NORA, envisioned to generate AP-problems automatically; such a generation is fundamental to close autonomic management loops and, so also, to realize self-driving networks. The NORA’s novelty lies in allowing the network administrator to express Goal Policies in NL and automatically transform them into AP-goals. NORA combines the AP-goals with the network status and network management tasks to generate AP-problems. NORA uses NLP as the translation technique and templates as the combination technique. To the best of our knowledge, we are pioneers in overcoming the interpretation gap between network management policies and AP-problems. Bridging this gap is fundamental for paving the self-driving network’s realization since network administrators do not need to spend time learning new policy formats or AP-notations when building up ACLs. In this way, they can focus on their core tasks. We implemented a NORA’s prototype and evaluated it using a Goal Policies dataset. Results show that NORA achieves high precision and spends a short-time on generating AP-problems. Consequently, we conclude that NORA is a promising solution to overcome barriers to using AP in self-driving networks.

The remainder of this paper is organized as follows. Section 2 describes the background and related work. Section 3 introduces NORA. Section 4 presents a prototype that instantiates NORA and its corresponding evaluation. Section 5 states concluding remarks and outlines future work.

## 2. Background and Related Work

Initially, this section introduces fundamental concepts to understand the relationship between self-driving networks and automated planning fully. Subsequently, it goes over some proposed strategies in diverse domains to transform policies to AP languages, mentioning relevant research work in the field. Finally, it explains the most commonly used methods to transform constrained natural languages into low-level network instructions.

### 2.1. Self-Driving Networks and Autonomic Control Loops

The exponential growth in the number of devices and users connected to networks places significant stress on current human-in-the-loop network management architectures. Thus, there is a rising interest in equipping networks with autonomous run-time decision-making capability by incorporating AI, ML, AP, big data, network analytics (NA), network telemetry combined with advances in networking (e.g., SDN, network functions virtualization and programmable data planes) to develop self-driving networks [14,15].

**Figure 1:**
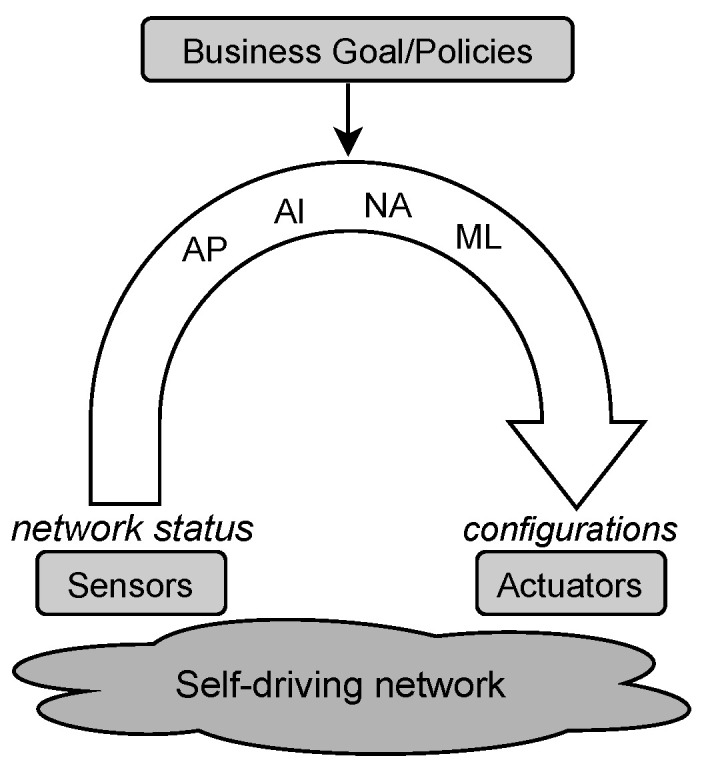
Closed ACL.

A self-driving network is an autonomous network where management control loops predict changes and adapt to user and traffic behavior without the intervention of a human operator [12,14]. Besides, according to [16,17] self-driving networks can measure, analyze and control themselves in an automated manner employing ACLs that react to changes in the environment by using sensors and actuators (see Figure 1). The sensors monitor the network operation (e.g., link occupancy or buffer size) via pull or polling techniques for getting information about its status. Algorithms based on AI, NA, and AP are useful for analyzing the sensed information and making decisions oriented to maintain any desired situation or overcome a problematic one. The actuators execute actions, such as enforce configurations in routing devices, in the managed network. Network policies play a crucial role in any self-driving network since every ACL must handle the network targeting to meet them [18].

Several proposals for accomplishing ACLs are available in the literature. For instance, the MAPE (Monitor-Analysis-Plan-Execution) introduced in late 2004 by IBM [19] (and applied to network management in [20,21]), and its extension FOCALE (Foundation Observation Comparison Action Learn rEason) [22] are classical ACL approaches for autonomic computing. In turn, CogMan [23] and C-MAPE [2] are ACL approaches for autonomic network management that employ a cognitive model for the loop operation. C-MAPE is also an extension of MAPE, where every function in the loop incorporates learning and inference functionalities. By its part, the Knowledge-Defined Networking paradigm [4] operates employing an ACL that combines ML, network analytics, and SDN.

### 2.2. Network Policies

Policies are guidelines and constraints to system management [24]. They represent service requirements, such as availability, response time, throughput, and security. According to [25] policies can be classified into Action Policy, Goal Policy, and Utility Function Policy. An Action Policy dictates the action that the Network Management System (NMS) should take whenever the system is in a given current state. Typically, an NMS based on Action Policies follows the structure IF(Condition) THEN(Action), where Condition specifies either a specific state or a set of possible states that all satisfy the given Condition. Note that the state that the NMS will reach taking the given action is not specified explicitly. Rather than specifying what to do in the current state S, a Goal Policy specifies how the NMS should behave when a single desired state σ, or one or more criteria that characterize an entire set of desired states happen. Goal Policies provide only a binary state classification: ’desirable’ and ’undesirable’ [26]. A Utility Function Policy is an objective function that expresses each possible state’s value. Utility Function Policies generalize Goal Policies.

In this paper, we work with Goal Policies because they are useful to feed the management control loops of self-driving networks as corroborated in [27,28]. When using Goal Policies to govern the behavior of self-driving networks, through autonomous NMS, the AP-based ACL is responsible for computing a network management task (or possibly a sequence of tasks or a workflow) that will cause the network to make a transition from the current state to some desired state. Rather than relying on a network administrator to explicitly encode rational behavior, the self-driving network generates rational behavior itself from the Goal Policy permitting greater flexibility and frees network administrator from the necessity of applying low-level commands at the underlying network, at the cost of requiring reasonably sophisticated AI-planning or, in overall, modeling AI-algorithms.

### 2.3. Automated Planning

AP is an AI field that automatically creates plans (set of possible actions) to go from an initial state (real-world situation) to a goal/target state. A planning problem involves these states and actions. A planning problem happens, for example, in a network operator that provides enhanced mobile broadband 5G slices by using Network Functions Virtualization (NFV), SDN, and ML, when an unexpected slice disruption occurs due to the NFV/SDN infrastructure outage or a security attack impacts negatively the congestion decisions made by a reinforcement learning algorithm. The disruption is the initial state. The goal state is to restore the slice as soon as possible, aiming at meeting a previously signed SLA (Service Level Agreement). The set of possible actions (describing real-world tasks) to use during the planning process are in a planning domain. Examples of actions are to replace the broken virtual network functions with their backups and re-build up the learning model.

Recently, it has been corroborated the feasibility of using AP to automate SDN management tasks and reduce the time required by network administrators to face network situations [3,29]. However, in such approaches, the network administrator still has to manually describe the network problem in AP notation, which is difficult to interpret without prior knowledge. To realize ACLs based on AP, the network itself should create without human intervention the planning problem.

### 2.4. Existing Work

Liu [5] proposed a mechanism to translate high-level objectives from the IT management domain into the goals of an AP-problem describing requirements for fault recovery. This mechanism uses rules to map fault expressions stated in the Domain Service Language (DSL) into the Planning Domain Definition Language (PDDL). However, the rules are attached to the IT management domain and, so their replication on the network management domain is hard. Other approaches [6,7] transformed user requests expressed in NL into PDDL for facing telecommunications services issues. Despite the use of NL and its integration with AP, these approaches present several drawbacks. First, NLP’s corpus is limited to requests related to the environmental early-warnings domain and, consequently, disregards models of Goal Policies. Second, the low-level configuration actions are specific for composing telecommunication services and leaves aside the network management tasks.

The works [8,9,30] introduced a framework to translate high-level policies expressed in CNL, into low-level flow rules for SDNs. The employed CNL follows a grammar of predefined regular expressions (regexes) representing terms of the network context such as “HTTP” and “FTP”. These works use Inductive reasoning for analyzing policy objectives and abductive reasoning for determining if the network infrastructure can accommodate the reasoned objectives during translations. Although this approach provides helpful grammar contributions regarding classification of the network context terminology, the inductive and abductive reasoning processes led directly from high-level to low-level commands disregarding the possibility of an AI algorithm to interpret network management regexes.

Tuncer et al. [11] proposed an approach for the automatic decomposition of High-Level Requirements (HLRs) to network management operations. This approach relies on developing a NorthBound Interface (NBI), including mapping functionality, that associates technical HLRs to the network operator’s services and functions that manage the network resources. This HLR-based approach performs the association through matching procedures to support operator-defined descriptors that encode distinct features and uniquely identify services and functions. In this approach, network administrators do not use NL and, consequently, they must fulfill the HLR format’s attributes.

Jacobs et al. [12] introduced an approach to translate network administrators policies expressed in NILE (i.e., an intermediate representation for network Intents) into network configurations. This approach uses a recurrent neural sequence-to-sequence learning model to extract Intents from NL and includes feedback from the network administrator for improving the learning process. Although this approach offers high accuracy in the translation process, it does not consider a closed-loop (requires feedback from network experts), pivotal for self-driving networks, and network administrators must learn NILE.

Riftadi and Kuipers introduced P4I/O [13], a framework that translates Intents into P4-programs using code-templates. Although the generated P4-programs offer excellent results for handling the network throughput, this framework requires that network administrators learn an extended version of NILE that adds custom actions for network tasks and does not support AI-based notations.

Widmer [31] proposed a state-machine-based refinement technique that uses a grammar for an Intent specification language and a parsing process to translate the intents to low-level blockchain selection policies abstracting underlying implementation details. This approach does not operate with policies expressed in NL nor explore AI-based notations.

The cited approaches share the following shortcomings. First, they require policies described in a particular syntax (e.g., CNL and Intents) that can be as hard to learn and interpret for network administrators as the AP notations are. Second, they assume a linear correspondence between high-level policies and network configuration tasks. In contrast, self-driving networks usually rely on AI algorithms to automatically and on the fly compute sequences of actions that carry out corrective and even preventive network configuration tasks to comply with high-level network management policies. In this sense, our proposal is a pioneer in transforming from high-level policies expressed in NL to AP notations in the network management domain. Bridging the interpretation gap between network management and AP facilitates the current intricate administrator work by closing AI-supported ACLs of self-driving networks.

## 3. NORA

In this section, we introduce how NORA operates at a high abstraction level. Furthermore, we explain in detail the architecture and modules composing NORA.

### 3.1. High-Level Operation

**Figure 2:**
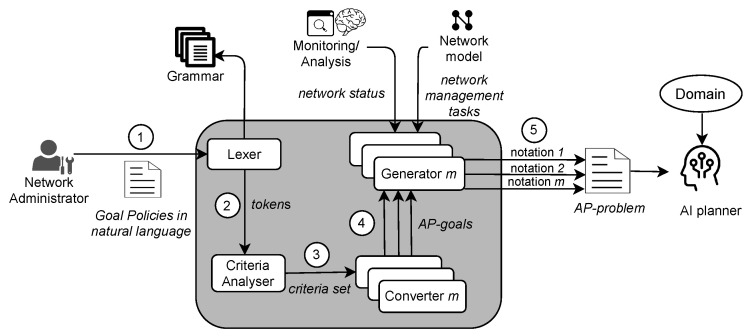
NORA architecture.

Figure 2 depicts NORA’s architecture conceived to generate AP-problems automatically. This paper argues that generating such problems is pivotal for achieving the self-driving network concept since they are essentials to close autonomic management loops. Henceforth, we focus on presenting how NORA translates Goal Policies expressed in NL into planning goals and combines them with network status and network management tasks to generate AI-planning problems. In turn, in this paper, we assume an external Monitoring and Analysis module, like the one proposed in [2,19], which provides the network status and a network model. The network model as described in [32] contains the management tasks necessary to realize the AP-based solutions.

**Figure 3:**
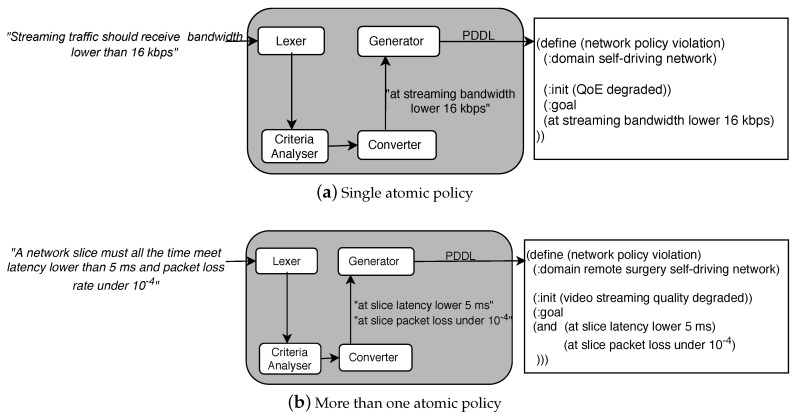
NORA—High-level operation.

A Goal Policy specifies either a single desired state σ, or one or more criteria that characterize a set of target states [25]. NORA operates with these policies because they are useful to express business goals without technical details as in the case of SLAs [8,30]. We model a Goal Policy as a 4-tuples P=<Target,Metric,Condition,Threshold>. In this model, Target is a binary <S|E>, where S denotes a network service and E denotes an endpoint (i.e., a network equipment or resource) or an end-user(s) (e.g., researchers working on a specific laboratory of a University). Metric denotes a network performance parameter measurable at services or endpoints. Condition denotes a boolean comparison adjective statement. Threshold denotes boundaries for the metric values. Thus, for using NORA, the network administrator must express policies as follows: “Streaming traffic should receive bandwidth lower than 16 kbps”, where Target: “Streaming traffic”, Metric: “bandwidth”, Condition: “lower than”, and Threshold: “16 kbps”. Goal Policies examples involving several network criteria, i.e., more than one atomic policy are: “HTTP services should receive bandwidth higher than 100 kbps and delay lower than 300 ms” and “A network slice must all the time meet latency lower than 5 ms and packet loss rate under $10^{-4}$”. Note that, as in the last examples, Goal Policies can be decomposed in several tuples <Target,Metric,Condition,Threshold>, leading to AP-problems with several planning goals (see Figure 3b).

From a high-level perspective and according to Figure 2, NORA operates as follows. First, the network administrator expresses a management policy by following the NL-based Goal Policy model. Second, the Lexer decomposes the policy in representative terms for the network management domain. These terms can be a word or a phrase, from now on called tokens. For example, “Voice over IP” and “Video streaming” are tokens representing a network service. Third, the Criteria Analyser forms a set of structured criteria where each element reflects an atomic policy involved in the incoming Goal Policy. Fourth, the Converter maps each element of the criteria set to a particular goal notation required by an AP-planner. Fifth, NORA builds up the AP-problem by combining the obtained planning goals with the network status and management tasks. Figure 3 exemplifies the output of NORA at steps 4 and 5 in PDDL notation for a Goal Policy with a single (Figure 3a) and several goals (Figure 3b). In the next subsections, we detail the NORA modules and how they interrelate to generate AP-problems from Goal Policies, network status, and network management tasks.

### 3.2. Lexer

This module receives management policies expressed by the network administrator in NL by following the Goal Policy model and extracts from them tokens. The Lexer builds up a matrix of tokens per each input policy as follows. First, it removes irrelevant terms to achieve faster tokens identification. Let us suppose a network policy defined for a remote surgery scenario as *P* = “A network slice must all the time meet latency lower than 5 ms and packet loss rate under $10^{-4}$”. In *P*, the terms removed would be “a”, “must”, “all the time”, and “meet”.

Second, the Lexer performs stemming to reduce words composing terms; e.g., in the raised policy “network slice” becomes “slice”, “lower than” becomes “lower” and “packet loss rate” becomes “packet loss”. Third, it carries out spell-checking to correct misspelled words or words damaged during stemming and compares remaining terms to expressions stored in a predefined domain grammar. Table 1 exemplifies our network management grammar based on [8]. Note that in Table 1 the Entity column corresponds to the 4-tuples defined for our policies model, i.e., Target,Metric,Condition,Threshold, and the Expression column corresponds to terms of network management argot classified under each entity type. The Connector entity in the last row refer to expressions that allow us to determine whether an input policy includes by several atomic policies, i.e., it involves more than one tuple (see detail in Section 3.3). Entities in the proposed grammar let categorize parts of an input policy instead of comparing with a set of specific policies; this offers flexibility to the extraction process.

**Figure 4:**
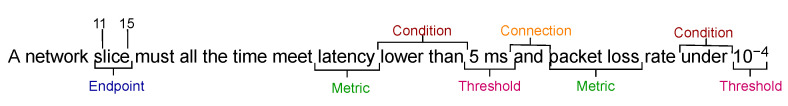
Identification of entities in a Goal Policy.

**Figure 5:**
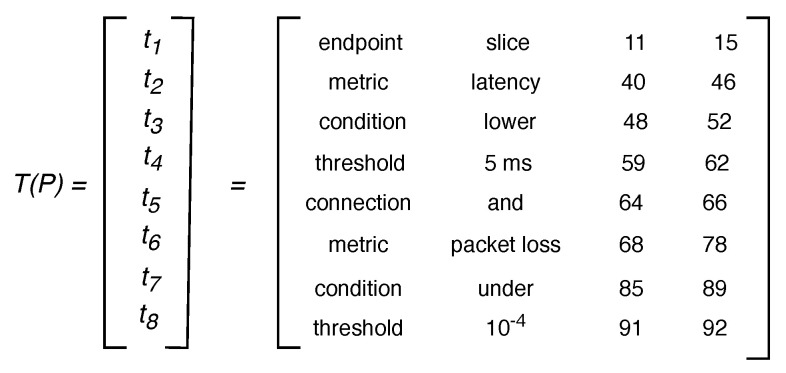
Matrix T for policy *P*.

Fourth, the Lexer marks as tokens the terms of the input policy matching grammar expressions and extracts their values and their positions in the original sentence. Figure 4 depicts the terms matched between the previous example policy P and the grammar (i.e., “slice”, “latency”, “lower”, “5 ms”, “and”, “packet loss”, “under”, “$10^{-4}$”), their corresponding entity type (i.e., endpoint, metric, condition, threshold, connection, metric, condition, threshold), and their start and end positions (e.g., the term “slice” begins and ends at positions 11 and 15, respectively). A further 4-tuples format allows to structure data extracted per token as t=<entityType,value,initialPosition,finalPosition>. Note that from each input policy *n* tokens can be marked, giving place to *n* tuples $t_{1},t_{2}...t_{n}$. We defined a 4 x *n* matrix, called *T*, to store the *n* tuples representing tokens derived from query policies, i.e., the rows of T are $t_{1},t_{2},...,t_{n}$. As an example, rows $t_{1},t_{2}...t_{8}$ in the T(P) matrix in Figure 5 correspond to the eight tuples for the tokens marked in the policy *P* presented in Figure 4. Note that the data for the first token marked in *P*, i.e., $t_{1}=<endpoint,slice,11,15>$, is the first row in T(P) and so on. Fifth, the Lexer sends T to the Criteria Analyser.

### 3.3. Criteria Analyzer

**Figure 6:**
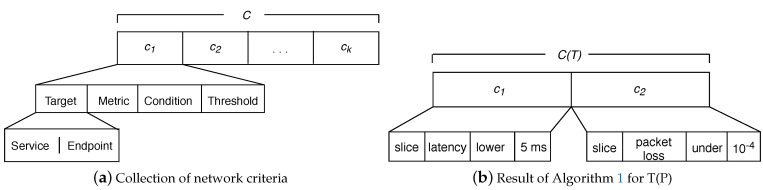
Criteria vs. Goal Policy Model.

This module receives each *T* matrix computed by the Lexer and delivers a corresponding set of criteria involved in the Goal Policy; Figure 6a shows how each network criteria follows our policies tuples model. Thus, the Criteria Analyzer transforms every T matrix in a collection of network criteria: i.e., $C=[c_{1},c_{2}...c_{k}]$, where $c_{i}$ is an atomic network management policy and *k* (i.e., the size of *C*) represents the quantity of atomic policies contained in an input policy. The tokens of type Connection (Table 1) in a policy allows obtaining the *k* value (see Equation (1)). For instance, in T(P) (Figure 5) there is one token of type Connection, i.e., $t_{5}$, which means that the raised policy P involves two atomic policies.

(1) k=Connection in T+1;

Algorithm 1 transforms the matrix *T* into the set *C*. Initially, this algorithm counts the number of Connection tokens in *T* (line 1). Then, it calculates *k* (line 2) and creates a *k*-size string vector (line 3) (e.g., in P, the values con = 1 and *k* = 2—from Figure 5 and Equation (1)—lead to $C=[c_{1},c_{2}]$). Finally, it fulfills each *c* by performing a cycle with *k* iterations, each time completing a network criteria $c_{i}$ conforming the set *C* (lines 4 to 14). In this cycle, Algorithm 1:

- Adds to $c_{1}$ the value of the endpoint or service with the minor initialPosition in the matrix *T* (lines 5 and 6). In the example, $t_{1}$ is the endpoint with the minor initialPosition (from Figure 5 initialPosition($t_{1}$) = 11); thus, at this step, $c_{1}=$ “slice”.
- Calculates proximity between tokens type metric and the previous selected token and adds the closest metric value to $c_{1}$ (lines 7 and 8). In the exemplified T(P), this choice is $t_{2}$, hence, current *c* becomes $c_{1}=$ “slice latency”. The algorithm runs a similar process for tokens of type condition and threshold (lines 9 to 12). In this way, in our example, $c_{1}=$ “slice latency lower 5 ms”.
- Marks as “used” appended tokens (line 13). Note that they can be appended to more than one $c_{i}$ when the quantity of a type of token in *T* is less than *k*. For instance, observe that in T(P) (Figure 5) $t_{1}$ is the only token of type target (i.e., service or endpoint), thus, its value, i.e., “slice”, is appended to $c_{2}$ although it was earlier appended to $c_{1}$. On the other hand, tokens of the matrix *T* can be discarded of the resulting set *C* if there is a (already “used”) token of the same type closest to the previous element of the tuple.

Once Algorithm 1 ends up, the Criteria Analyzer sends the criteria set (*C*) to the Converter. In the example, the transformation of T(P) after executing Algorithm 1 is $C=[c_{1},c_{2}]$, where $c_{1}=$“slice latency lower 5 ms” and $c_{2}=$“slice packet loss under 10-4” (see Figure 6b).


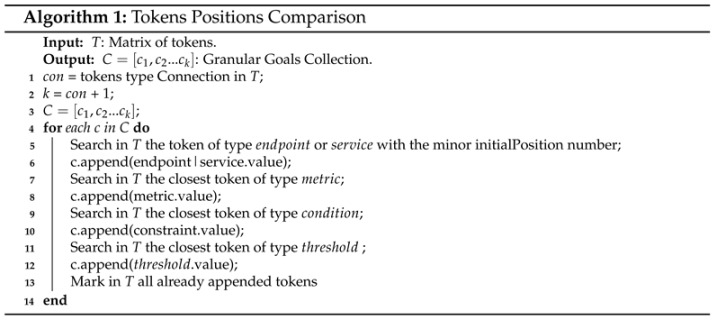


### 3.4. Converter

This module maps the elements of *C* (set of criteria: $c_{1},c_{2}...c_{k}$) to a particular AP-goal notation (e.g., PDDL) that is compatible with a specific planner (e.g., Simple Hierarchical Ordered Planner [33] or Hierarchical Task Planner [34]). This planner can be used for closing the autonomic management loop. As there are multiple planning goal notations, the Converter defines a repository of *m* mapping functions (or converters) and a selection function that calls the appropriate mapping for the target AP-problem notation.

To exemplify the Converter, we overview its operation when using PDDL and STRIPS. In PDDL, the problem file reserves a piece of code for specifying goals. Listing 1 shows the syntax that the PDDL must generate. STRIPS defines problems using a boolean value function that allows describing the problem by logical conditions and specifies the problem goal as “things that we want to be true”. Listing 2 shows that the STRIPS converter must generate the goal by placing the policy’s Condition followed by its remaining parameters (in brackets). This condition can be true or false.

**Listing** **1**. AI-planning goals in PDDL.

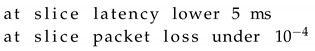

**Listing** **2**. AI-planning goals in STRIPS

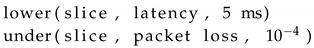

### 3.5. Generator

From a general perspective, a planning-problem definition involves deciding what actions to execute given a goal and an initial state [1]. Thus, the primary entries for describing a problem in AI-planning based solutions are predicates defining an initial state, a problem goal, and (a reference to) a set of tasks (atomic or composed). NORA gets the problem goals automatically from Goal Policies expressed in NL. In turn, in NORA, the initial state corresponds to the network status obtained from an external Monitoring and Analysis module available in solutions running MAPE [19] or C-MAPE [2] ACLs. NORA assumes this loop delivers network status like “streaming quality degraded” and “QoE degraded”. NORA retrieves the network management tasks from an external network model like the YANG-based and SDN-centered proposed in [32]. An example of a network management task is “scale up a VNF” or “scale down a VNF”. We do not detail about initial state and management tasks because it is out of the scope of this paper. We address them to get a closed ACL based on AI-planning techniques in future work. Summarizing, the Generator builds up an AI-planning problem by combining a Goal Policy received from the Converter, the network status, and management tasks (along with their preconditions and effects). The implementation of this module follows a templates-based approach. Since NORA needs to build up problems into different AI-planning notations, similar to the Converter, *m* Generators are needed. Each one uses the corresponding template for the particular target notation.

**Figure 7:**
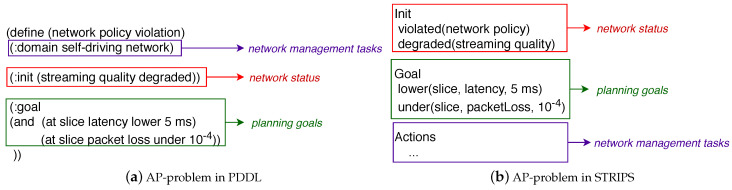
AP-problems.

Figure 7a shows an AP-problem described with the PDDL templates proposed in [35,36]. In such templates, the problem attributes follow a schema-like representation including mainly: (i) name, i.e., a string used to identify the planning problem—in the example “Network Policy Violation”, (ii) domain where actions (i.e., network management tasks) are specified, (iii) initial state is the network status; and (iv) goal state containing one or several atomic goals that correspond to the Goal Policy in AP notation. Figure 7b shows an STRIPS-based AP-problem that includes the sections: Init, Goal, and Actions. The Init section corresponds to the network status. The Goal is the translated Goal Policy. The Actions are the network management tasks that the AP planner will use to achieve the Goal.

NORA sends the resulting AP-problem to the planner responsible for computing a management plan intended to obtain a closed network management ACL. An AP-based management plan is a sequence of management tasks that, once enforced in the underlying network, cause the network to go from the current status to another that meets the translated Goal Policy; recall, it is initially expressed in NL by the network administrator and translated to AP notation by NORA.

## 4. Evaluation

This evaluation aims to assess and discuss NORA’s performance when generating AP-problems from Goal Policies, network status, and network management tasks. This section initially introduces the prototype of NORA and the Goal Policies dataset used in the tests. This section then describes the performance metrics assessed, namely Precision and Processing Time. Finally, this section presents and discusses the NORA’s evaluation results.

### 4.1. Prototype

**Figure 8:**
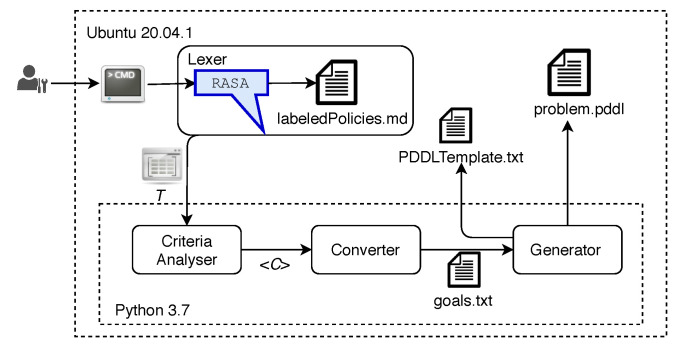
NORA prototype.

Figure 8 depicts the prototype of NORA. The Lexer module was instantiated by using Rasa 1.10.0 [37], an ML-based NLP tool that allows understanding and manipulating NL for extracting tokens [38]. We used Rasa because a recent comparative study on NLP services’ performance demonstrated that it overcomes similar tools, such as LUIS [39] and Lex [40], in terms of adaptability and customization thanks to its open-source nature [41]. The Linux Command Line is the user interface of NORA.

**Figure 9:**
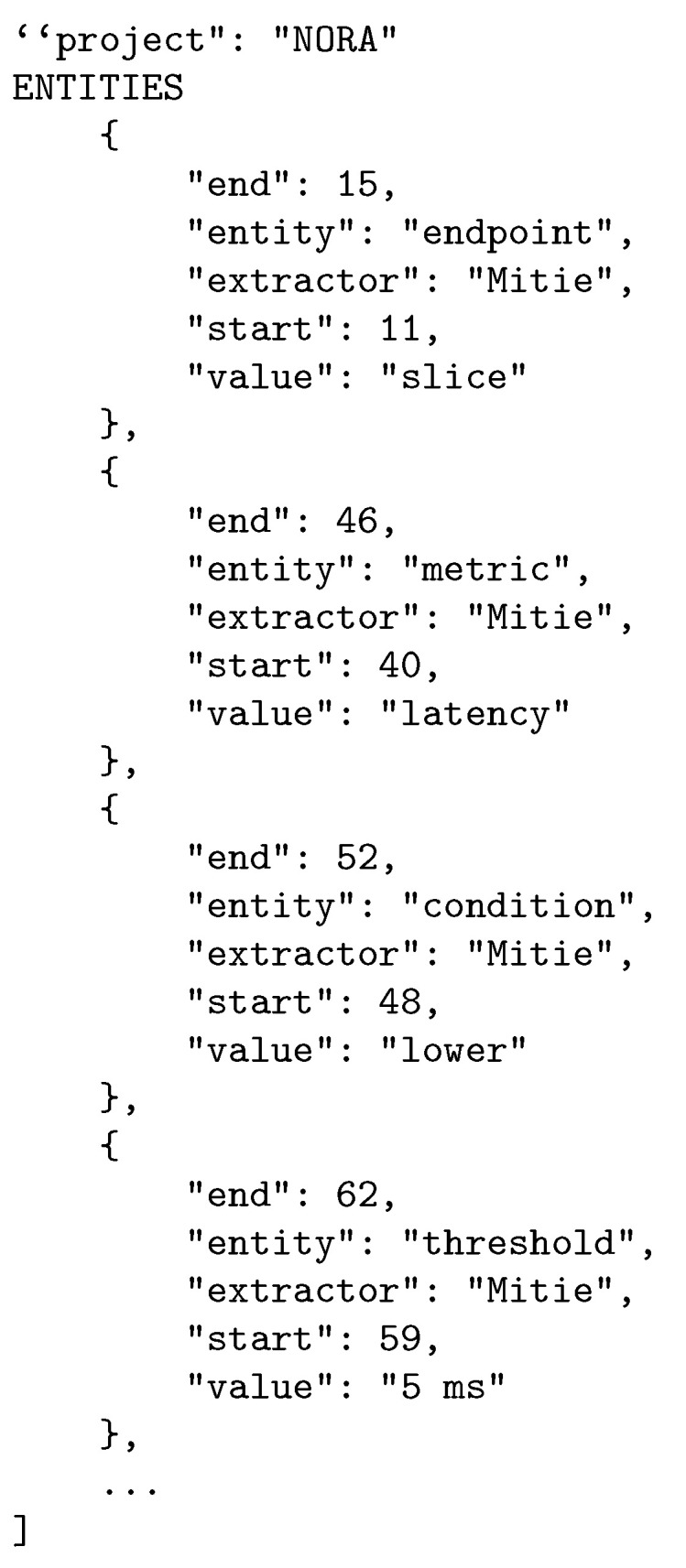
Lexer output.

Figure 9 presents as example the tokens extracted by the Rasa-based Lexer when processing the Goal Policy *P* = “A network slice must all the time meet latency lower than 5 ms and packet loss rate under $10^{-4}$”. The data retrieved per policy are: (i) end, the position of the last character of the token in the policy, (ii) entity, the type of token according to the Grammar, (iii) extractor, an identifier for the ML-based engine used in the learning and extraction processes, (iv) start, the position of the first character of the token in the policy; and (v) value, the token itself as it appears in the policy.

We implemented the modules Criteria Analyser, Converter, and Generator as Python programs. These programs were integrated into the Rasa-based Lexer by inheriting from its Action class [42]. Specifically, we developed a Custom Action that implements Algorithm 1 responsible for mapping Goal Policies into AP-goals in PDDL notation as in Listing 2 and generating PDDL-problems by filling out PDDL-problem templates [43]. These PDDL-problems stored in system files, jointly with a well-defined planning domain file, are enough input for executing an AI Planner, such as the STRIPS engine (Standford Research Institute Problem Solver) [44], responsible for automatically generating the corresponding management plan. For the sake of experimentation, we have used the PDDL notation for specifying the goals and problems of AP. However, it is noteworthy that the Converter and Generator modules can be implemented for NORA operates with other AP-notations (e.g., STRIPS and Action Description Language).

### 4.2. Goal Policies Dataset and Lexer Tuning Up

We created a Goal Policies dataset to tune up the NLP-based Lexer that allows NORA to learn how a network management policy is usually written and, so, to identify and extract tokens automatically; the NORA’s precision heavily depends on the Lexer success. This dataset was built as follows. First, we collected 250 Goal Policies from 20 network management researchers. Second, we labeled each network management term of each policy with the corresponding entity type (i.e., service, endpoint, metric, condition, and threshold) according to our grammar (Table 1) and stored them in a plain text file (“labeledPolicies.md” in Figure 8) that Rasa is able to interpret as training data. For instance, the term “streaming” was labeled as service. Note that a term written in different ways -or with synonyms- like “P2P”, “Peer to Peer” or “Peer-to-Peer” leads to the same label; in this example service. Third, we took the labeled policies as a base corpus for NORA and automatically generated further policies to obtain a dataset with 1000 Goal Policies. For this, we performed random combinations of terms for services or endpoints, metrics, conditions and thresholds, and added complementary expressions to complete phrases, e.g., “On demand, network infrastructure must be configured for...”, “... compared with other services...”, “NORA, the network must...”.

We tuned up the Lexer module by using the cross-validation technique that allows using all available data for training and testing by splitting it into *k* number of groups [45,46]; we used k=10. Once tuned up, the NLP-based Lexer identified with high precision the tokens: Service(92.6%), Metric(99.3%), Endpoint(93.2%), and Constraint(90%). Conversely, this module identified with moderate precision (70%) the tokens of type Threshold; to increase this precision is necessary to add into the Goal Policies dataset more policies containing the label Threshold. Table 2 highlights in blue color as example some failures on the Lexer’s operation, i.e., tokens not identified or wrongly classified.

### 4.3. Performance Metrics

**Figure 10:**
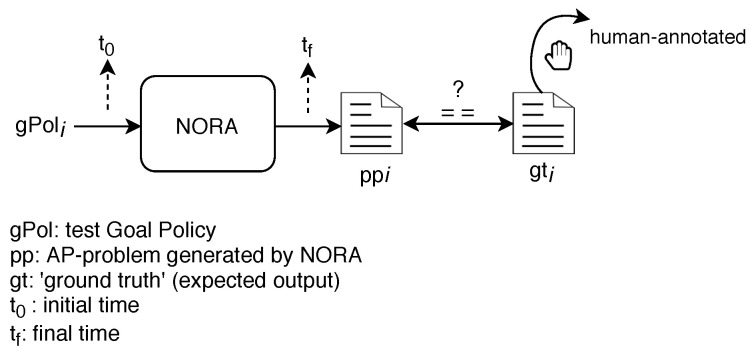
Test metrics.

We evaluated NORA using the Precision and Processing Time performance metrics. Precision allows measuring whether NORA produces precise translations, meaning an AP-problem generated by NORA includes the Goal Policy, network status, and network management tasks appropriate. High Precision is a mandatory requirement to push the NORA’s adoption in self-driving networks. We measured Precision as follows (see Figure 10). First, we created 1000 testing tuples <gPol,gt> where gPol represents an incoming Goal Policy and gt its corresponding ground truth. Each $gt_{i}$ is the expected PDDL-problem file given the $gPol_{i}$ and assuming as known the network status and network management tasks. Second, we computed NORA precision by using Equation (2) where agr (agreement) is a boolean variable. agr is equal to 1 when the planning problem generated by NORA ($pf_{i}$) matches the ground truth ($gt_{i}$) in terms of their textual content and syntax. Otherwise, agr is equal to 0. In turn, *n* refers to the total number of test query policies, and the summation *A* represents the overall NORA precision.

(2) A=∑i=1nagrintinyagr=1if ppi==gti0in other case

Processing Time allows measuring the speed of NORA for generating AP-problems. NORA’s quickness is crucial when considering its adoption in self-driving networks because ACLs should address undesired network states (detected and triggered by Monitoring/Analysis modules) on-the-fly before the network instability expands and affects the Quality of Experience. We measured ProcessingTime as the time elapsed since NORA receives a test query Goal Policy until it generates the corresponding AP-problem file, i.e., from $t_{0}$ until $t_{f}$ in Figure 10.

### 4.4. Results and Analysis

**Figure 11:**
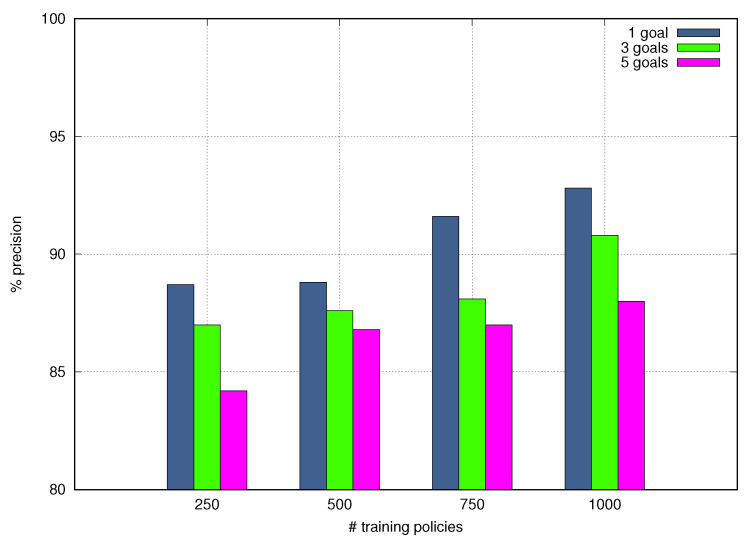
Precision vs. Training Policies.

Figure 11 depicts Precision values achieved by NORA as a function of the number of training policies and the granular goals involved in each test query Goal Policy. The NORA’s Precision increases when the number of training policies rises; meaning that, as expected, a large Goal Policy dataset leads to improve the Lexer behavior regarding tokens identification. The Precision of NORA decreases when the number of granular goals expressed in the test query Goal Policies increases; meaning that complex policies hinder the NORA’s behavior. In particular, NORA obtained the highest Precision, around 92.8%, with the dataset including 1000 Goal Policies and with a single granular goal per test query Goal Policy. NORA got the worst Precision, about 84.2%, with the dataset including 250 Goal Policies and with five granular goals per test query Goal Policy. The high-Precision obtained by NORA shows it is a promising solution to generate the AP-problems needed to close the autonomic management loops that allow realizing self-driving networks.

**Figure 12:**
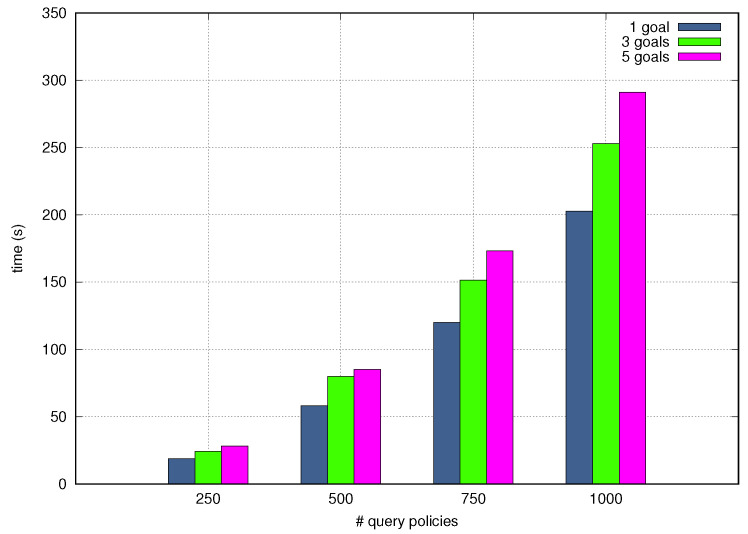
End-to-end processing time by granular goals.

**Figure 13:**
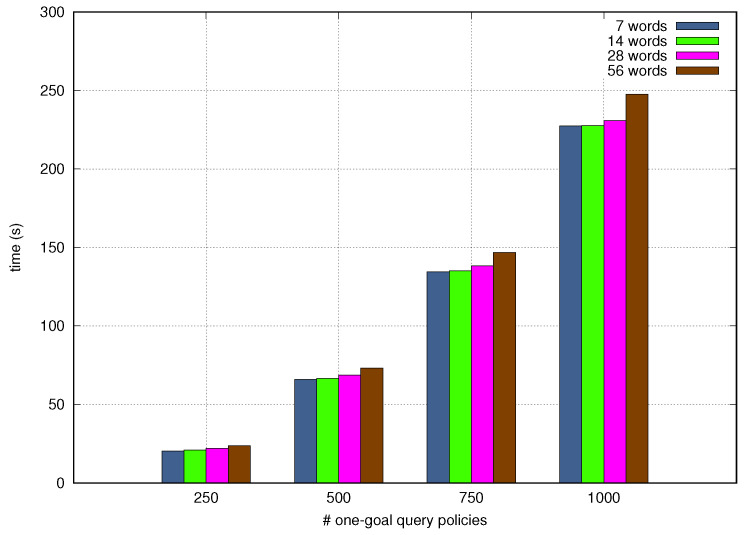
End-to-end processing time by policy length.

Figure 12 and Figure 13 depict the Processing Time as a function of the quantity of test query Goal Policies incoming one after another and the number of granular goals and words per tets query Goal Policy. The NORA’s Processing Time increases when the number of incoming policies, goals, and words per policy grow up, although the last criteria, i.e., words per policy, slightly alters the Processing Time. In particular, NORA obtained the worst Processing Time, around 290 seconds, when simultaneously translating 1000 policies with five goals each. NORA got the best Processing Time, about 20 seconds when simultaneously translating 250 policies with 1 goal per policy. The low-Processing Time obtained by NORA shows it allows closing the autonomic management loops quickly which is fundamental in the context of self-driving networks.

Since NORA is an AP-problems generation approach with no precedents in the networking domain, there is no conventional method to perform a direct comparison. Therefore, in the next lines, we compare NORA to HAUTO [7], a framework that includes an NLP-based module for transforming NL and environmental early warning information into PDDL problems. NORA achieved in average a Precision (92.8%) slightly lower than the obtained by HAUTO (94.4%). We can improve the NORA’s Precision by increasing the dataset size and the grammar expressions; this is part of our next research steps. NORA when processing a Goal Policy including five goals got a Processing Time (290 milliseconds) equal to the achieved by HAUTO for analyzing a user requirement phrase and generating the PDDL problem file. This preliminary benchmark corroborates the NORA results are promising to put self-driving networks into reality.

### 4.5. Languages Extensibility

We conceive NORA to operate with grammar and corpus defined in the English language since it is the most common language used in the network management area; for instance, network operators and equipment vendors usually specify Service Level Agreements (SLAs) and Command Line Interfaces (CLIs) in English. Therefore, we describe and evaluate the NORA’s components using English sentences. However, it is remarkable that the NORA’s architecture does not need changes to support Goal Policies specified in diverse languages.

NORA can be adapted to deal with Goal Policies expressed in diverse languages by following the next steps. First, redefining the network management grammar (Table 1) in the new language. Second, collecting Goal Policies in such a language; these policies are the basis for the corpus generation. Third, labelling the new-language Goal Policies corpus according to the grammar expressions. Fourth, tuning up the Lexer with the labelled policies (see Figure 8, labeledPolicies.md file).

## 5. Conclusions

This paper introduced NORA, an approach that automatically generates AP-problems by transforming Goal Policies expressed in NL into AP-goals and combining them with both the network status and the network management tasks.The evaluation results showed that NORA achieves a precision near 92.8% and spends around 0.084 seconds on generating AP-problems, which evinces our approach is useful to overcome barriers to using AP to realize autonomic management scenarios that are pivotal for accomplishing self-driving networks. For future work, we plan to enhanced NORA by supporting another kind of network management policy. Moreover, we intend to extend the NORA’s scope in order to achieve an autonomic management solution.

## Figures and Tables

**Table 1:** Network management grammar.

Entity	Expression
Service	VoIP, Streaming, HTTP, FTP, SMTP, P2P...
Endpoint	gateway, database, slice, VM, CPU, client, user...
Metric	bandwidth, delay, throughput, jitter, load, latency, packet loss...
Condition	more, high, higher, up, over, exceed, not under,...
	equal, like, even, same, similar,...
	less, lower, not exceed, down, below, under,...
Threshold-unit	ms, s, kbps, GB, GHz, %...
Connection	and, also, as well as, or...

**Table 2:** Lexer tokens identification vs. Ground-Truth.

Tokens Ground-Truth Labeled by Experts (Expected)					
ID	Service	Metric	Endpoint	Constraint	Threshold
1	- -	load	CPU/VM’s	not exceed/not be under	80%/20%
2	streaming	bandwidth	- -	higher than	20 Mbps
3	streaming	bandwidth	- -	higher than	20 Mbps
4	- -	latency	client B	less than	10 ms
5	download	- -	professors	no more than	1,000,000 MB per week
**Lexer Tokens Identification**					
ID	Service	Metric	Endpoint	Constraint	Threshold-unit
1	- -	load	CPU/ - -	not exceed/not be under	80%/20%
2	streaming	bandwidth	- -	higher than	20 Mbps
3	streaming	bandwidth	- -	higher than	20 Mbps
4	- -	latency	client	less than	10 ms
5	download	- -	professors	more than	- -

## Data Availability

The Goal Policies dataset is available at https://github.com/arodriguezvivas10/GoalPolicies (accessed on 3 March 2021).

## Abbreviations

The following abbreviations are used in this manuscript:

ACL	Autonomic Control Loop
AI	Artificial Intelligence
AP	Automated Planning
CNL	Controlled Natural Language
DSL	Domain Service Language
HLR	High-Level Requirement
MAPE	Monitor-Analysis-Plan-Execution
ML	Machine Learning
NA	Network Analytics
NBI	NorthBound Interface
NFV	Network Functions Virtualization
NL	Natural Language
NLP	Natural Language Processing
NMS	Network Management System
PBNM	Policy-based Network Management
PDDL	Planning Domain Definition Language
SDN	Software-Defined Networks
SLA	Service Level Agreement
